# Drug-resistant tuberculosis: challenges and opportunities for diagnosis and treatment

**DOI:** 10.1016/j.coph.2018.05.013

**Published:** 2018-10

**Authors:** Anastasia Koch, Helen Cox, Valerie Mizrahi

**Affiliations:** 1 SAMRC/NHLS/UCT Molecular Mycobacteriology Research Unit, DST/NRF Centre of Excellence for Biomedical TB Research and Wellcome Centre for Clinical Infectious Diseases Research in Africa, University of Cape Town, South Africa; 2 Institute of Infectious Disease and Molecular Medicine and Division of Medical Microbiology, Department of Pathology, Faculty of Health Sciences, University of Cape Town, South Africa

## Abstract

With an estimated incidence of 490 000 cases in 2016, multidrug resistant tuberculosis (TB), against which key first-line anti-tuberculars are less efficacious, presents major challenges for global health. Poor treatment outcomes coupled with a yawning treatment gap between those in need of second-line therapy and those who receive it, underscore the urgent need for new approaches to tackle the scourge of drug-resistant TB. Against this background, significant progress has been made in understanding the complex biology of TB drug resistance and disease pathogenesis, and in establishing a pipeline for delivering new drugs and drug combinations. In this review, we highlight the challenges of drug-resistant TB and the ways in which new advances could be harnessed to improve treatment outcomes.

## Highlights

• Management of tuberculosis is complicated by high levels of drug resistance in some regions of the world.
• Increasingly, molecular diagnostics are being used for resistance detection to certain first-line anti-TB drugs.
• Genotype-phenotype relationships for resistance to other drugs are complex making DST by molecular methods challenging.
• Individualized approaches to MDR-TB treatment management may help to minimize the development of further resistance.
• Individualized approaches to MDR-TB treatment management may help to minimize the development of further resistance.

## Graphical abstract

**Figure:**
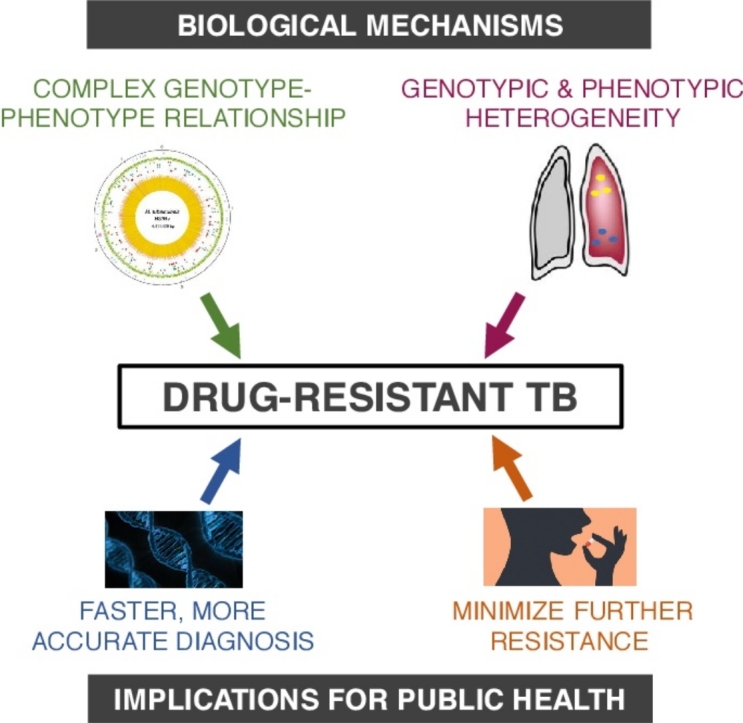

**Current Opinion in Pharmacology** 2018, **42**:7–15

This review comes from a themed issue on **Anti-infectives**

Edited by **Timothy Egan** and **Digby Warner**

For a complete overview see the Issue and the Editorial

Available online 6th June 2018

**https://doi.org/10.1016/j.coph.2018.05.013**

1471-4892/© 2018 The Authors. Published by Elsevier Ltd. This is an open access article under the CC BY license (http://creativecommons.org/licenses/by/4.0/).

## Introduction

Claiming an estimated 1.7 million lives in 2016, tuberculosis (TB) is now the leading cause of death worldwide from a single infectious agent [1]. Of the 10.4 million incident cases of TB in 2016, an estimated 600 000 were rifampicin (RIF)-resistant (RR-TB), of which 490 000 were multidrug-resistant (MDR-TB), defined as resistant to isoniazid (INH) and RIF, with or without resistance to other first-line drugs. Extensively drug-resistant TB (XDR-TB), defined as MDR-TB with resistance to any fluoroquinolone and at least one of the second-line injectables, amikacin, capreomycin or kanamycin, accounted for 6.2% of the estimated incidence of MDR-TB in 2016. However, the treatment gap is vast, as evidenced by the fact that the number of MDR/RR-TB cases started on treatment that year was less than a quarter of the estimated incidence of MDR/RR-TB [1]. To further aggravate the problem, the currently recommended therapeutic regimens for drug-resistant TB (DR-TB) have poor efficacy and tolerability. As a result, treatment outcomes are poor, with success rates of 54% and 30% being reported for MDR-TB and XDR-TB treatment, respectively, based on 2014 cohorts [1]. These grim statistics underscore the urgent need for improved access to both diagnosis and effective treatment for all forms of DR-TB. In this article, we provide a brief overview of key challenges in the diagnosis and clinical management of DR-TB, and describe how advances in understanding the biology of TB drug resistance and disease pathogenesis are being brought to bear on addressing this major global health challenge.

## The complex genetics of TB drug resistance

*Mycobacterium tuberculosis* (Mtb), the causative agent of TB, is an obligate pathogen that is thought to have co-existed with its human host for millions of years [2^••^]. The same features of the metabolism and physiology of Mtb that enable it to persist quiescently for years within the human host present formidable challenges for effective chemotherapy [3] (Box 1). For the purposes of this review, it is important to distinguish drug *resistance* — the heritable ability of an organism to resist the effects of an antibiotic to which its parent was susceptible — from drug *tolerance* and *persistence*, which allow transient survival of an organism at concentrations of an antibiotic that would otherwise be lethal (Box 1) [4^••^].

Box 1 **Drug resistance, drug tolerance and persistence.**

Specific features of the tubercle bacillus present challenges for TB drug efficacy [3,62]. The complex, lipid-rich cell wall forms a barrier to drug penetration [63] and provides a mechanism to dysregulate the host immune response [64]. Mtb adapts physiologically to the various hostile environments encountered during infection [62], and by entering into states of slow, or no growth, it becomes refractory to antibiotics that act on cellular processes essential for growth [65]. Efflux systems are also thought to mitigate the efficacy of certain drugs by lowering their intracellular concentrations [66]. Changes in Mtb physiology lead to a mixed population of bacilli in a variety of metabolic states, which complicates drug treatment [50].

Differential responses of a bacterial population to drug treatment can arise from drug resistance, drug tolerance or persistence; all three mechanisms are thought to apply in TB. Balaban and colleagues [4^••^] have proposed framework for distinguishing these mechanisms on the basis of MIC and ‘minimum duration of killing’ (MDK) values, where the MIC_99_ represents the minimum concentration of drug required to kill 99% of the bacterial population, whereas the MDK_99_, represents the minimum time required to kill 99% of a population. **Drug resistance** is heritable, and usually occurs as a result of a mutation in the gene encoding either the target of the drug or the enzyme which activates the prodrug (Table 1). Resistance results in a net decrease in the effectiveness of a drug, and an observable increase in the MIC_99_. Cells that are able to transiently survive exposure to concentrations of an antibiotic that would otherwise be lethal display phenotypic **drug tolerance** [4^••^]. Drug tolerance can occur through a variety of mechanisms such as slow growth and is a population-wide phenotype. Phenotypically drug-tolerant populations have similar MICs to those of fully susceptible populations, but the MDK_99_ value is significantly higher. **Persistence** is similar to phenotypic drug tolerance in that it describes transient survival in the presence of inhibitory concentrations of antibiotic, but is distinct in that only a small percentage of the population displays this phenotype. Persistence is characterised by bi- or multi-phasic kill kinetics, where the majority of the population is killed rapidly by the antibiotic, whereas the persisters are killed much more slowly. In this case, the MIC_99_ and MDK_99_ values are similar to a susceptible population, but the MDK_99.99_ value is much higher than for a susceptible population [4^••^].

Alt-text: Box 1

In many bacterial pathogens, horizontal gene transfer (HGT) plays a major role in the acquisition of drug resistance determinants. However, HGT is thought to be negligible in Mtb [2^••^]; instead, drug resistance is mediated by single nucleotide polymorphisms (SNPs), multinucleotide polymorphisms, indels and rearrangements in chromosomal genes that encode drug targets; enzymes that metabolise prodrugs to their active forms, or drug efflux systems [5, 6] (Table 1). For some TB drugs, such as RIF [[2^••^],^9^], the genotype–phenotype relationship with respect to resistance is well-established whereas for others, the association is less clear. Moreover, in the case of RIF, the range of resistance-conferring mutations is quite restricted, whereas a much wider range of mutations can confer resistance to pyrazinamide (PZA), being scattered across the entire *pncA* gene [7^•^].

**Table 1:** Drugs used for the treatment of TB as classified by the WHO [38,51,52] and Mtb genes in which resistance-conferring mutations are commonly observed

Drug	Chemical class	Mechanism of action	Mtb gene/s in which DR-conferring mutations are commonly observed^a^	Included in WHO-endorsed molecular diagnostics	References
**First-line oral drugs**^b^					
Isoniazid^c^	Pyridine	Inhibition of mycolic acid synthesis	*katG*, *inhA*	Yes: MTBDR*plus* (V1.0 and V2.0) and Nipro NTM + MDRTB	[53, 54, 55]
Pyrazinamide^c^	Pyrazine	Disruption of energy homeostasis; inhibition of trans-translation and coenzyme A biosynthesis	*pncA*, *rpsA*, *panD*	No	[7^•^,^53^,^54^]
Ethambutol	Ethylenediamine	Inhibition of arabinogalactan biosynthesis	*embB*, *ubiA*	Yes: *embB* in MTBDR*sl* (V1.0 only)	[53, 54, 55]
Rifampicin	Rifamycin	Inhibition of RNA synthesis	*rpoB*	Yes: GeneXpert Mtb/RIF, MTBDR*plus* (V1.0 and V2.0) and Nipro NTM + MDRTB	[53, 54, 55]

**Group A: Fluoroquinolones**^d^					
Levofloxacin	Fluoroquinolone	Inhibition of DNA synthesis	*gyrA*, *gyrB*	Yes: *gyrA* MTBDR*sl* (V1. 0 and V2.0) *gyrB* in V2.0 only	[53,54,56]
Moxifloxacin	Fluoroquinolone	Inhibition of DNA synthesis	*gyrA, gyrB*	Yes: *gyrA* MTBDR*sl* (V1. 0 and V2.0) *gyrB* in V2.0 only	[53,54,56]
Gatifloxacin	Fluoroquinolone	Inhibition of DNA synthesis	*gyrA, gyrB*	Yes: *gyrA* MTBDR*sl* (V1. 0 and V2.0) *gyrB* in V2.0 only	[53,54,56]

**Group B: second-line injectable drugs**					
Kanamycin	Aminoglycoside	Inhibition of protein synthesis	*rrs*, *eis*, *whiB7*	Yes: *rrs* in MTBDR*sl* (V1. 0 and V2.0) *eis* in V2.0 only.	[53,54,56]
Amikacin	Aminoglycoside	Inhibition of protein synthesis	*rrs*, *eis*, *whiB7*	Yes: *rrs* in MTBDR*sl* (V1. 0 and V2.0) *eis* in V2.0 only.	[53,54,56]
Capreomycin	Aminoglycoside	Inhibition of protein synthesis	*rrs*, *tlyA*	Yes: *rrs* MTBDR*sl* (V1. 0 and V2.0)	[53,54,56]
Streptomycin	Aminoglycoside	Inhibition of protein synthesis	*rpsL*, *rrs*, *gidB*	Yes: *rrs* MTBDR*sl* (V1. 0 and V2.0)	[53,54,56]

**Group C: other core second-line agents**					
Clofazimine	Riminophenazine	Disruption of energy metabolism	*Rv0678*	No	[12^•^,^53^,^54^]
Linezolid	Oxazolidinone	Inhibition of protein synthesis	*rrl, rplC*	No	[53,54]
Cycloserine	d-Alanine analogue	Inhibition of peptidoglycan biosynthesis	*alr, ddl, cycA*	No	[53,54,57]
Terizidone	d-Alanine analogue	Inhibition of peptidoglycan biosynthesis	Potentially similar to cycloserine	No	[53,54]
Ethionamide^c^	Pyridine (thioamide)	Inhibition of mycolic acid biosynthesis	*etaA/ethA, ethR, inhA*	No	[53,54]
Prothionamide^c^	Pyridine (thioamide)	Inhibition of mycolic acid biosynthesis	Potentially similar to ethionamide	No	[53,54]

**Group D: Add-on agents (do not form part of the core regimen for MDR-TB)**					
Pyrazinamide^c^	Pyrazine	Disruption of energy homeostasis; inhibition of trans-translation and coenzyme A biosynthesis	*pncA, rpsA, panD*	No	[7^•^,^53^,^54^]
Ethambutol	Ethylenediamine	Inhibition of arabinogalactan biosynthesis	*embB, ubiA*	Yes: *embB* in MTBDR*sl* (V1.0 only)	[53,54,55]
High-dose isoniazid	Pyridine	Inhibition of mycolic acid synthesis	*katG, inhA*	No	[53,54]
Bedaquiline	Diarylquinoline	Inhibition of ATP homeostasis	*atpE*, *Rv0678*	No	[12^•^,^53^,^54^]
Delamanid^c^	Nitroimidazole	Complex mechanism, including inhibition of mycolic acid biosynthesis	*ddn, fdg1*	No	[53,54]
Amoxicillin and clavulanate	Penicillin/β-lactam	Inhibition of cell wall biosynthesis		No	[53,54,58]
Para-aminosalicylic acid^c^	Salicylate	Inhibition of folic acid and thymine nucleotide metabolism	*thyA, dfrA, folC, ribD*	No	[53,54]
Thioacetazone^c^	Thiosemicarbazone	Inhibition of mycolic acid biosynthesis	Potentially *ethA*	No	[53,54,59,60]
Imipenem and cilastatin	Carbapenem	Inhibition of cell wall biosynthesis	Potentially Rv2421c-Rv2422	No	[53,54,61]
Meropenem and clavulanate		Inhibition of cell wall biosynthesis	Potentially Rv2421c-Rv2422	No	[53,54,61]

a See [26] for a list of specific mutations and associated levels of resistance.

b Rifabutin could be considered if the Mtb strain is resistant to RIF but susceptible to rifabutin [51].

c Prodrug.

d TB antibiotic groupings as defined by the WHO policy recommendations in 2016, which focusses on treatment of DR-TB [38,51,52].

Cross-resistance can occur to TB drugs within the same class as well as between classes. For example, mutations in *gyrA* and *gyrB* can result in cross-resistance to multiple fluoroquinolones [10]. Likewise, mutations in *rpoB* that confer resistance to RIF can result in cross-resistance to other rifamycins. In a sobering example of cross-resistance with potential implications for the management of DR-TB, a mutation in a transcriptional regulator, Rv0678, was shown to result in cross-resistance of Mtb to clofazimine — a leprosy drug used in DR-TB treatment — and bedaquiline, a drug recently approved for the treatment of MDR-TB [11], through upregulation of a multi-substrate efflux pump [12^•^]. Curiously, a markedly higher prevalence of resistance-associated variants in *Rv0678* was found in MDR-TB patients with no evidence of prior use of clofazimine or bedaquiline than in non-MDR-TB patients [13] suggesting an association with prior TB drug exposure. Although the underlying driver/s remains unclear, these findings highlight the formidable range of mechanisms that Mtb can engage to evade drug pressure which complicates the design new therapeutic regimens for DR-TB.

## Diagnosis of TB drug resistance: from culture to whole-genome sequencing

Traditionally, drug susceptibility testing (DST) for TB has been conducted phenotypically using culture-based methods; however, these have a number of caveats (Box 2). More recently, molecular diagnostics that detect mutations associated with resistance to TB drugs have been implemented in some settings [1]. The major advantages of these diagnostic modalities are speed of detection of resistance and ease of use. The two most widely used molecular tests are the Xpert MTB/RIF cartridge-based system (Cepheid), and Hain line probe assay (LPA) (Hain Lifescience). Currently, Xpert MTB/RIF only detects RIF resistance caused by the most common mutations in the Rifampicin Resistance Determining Region (RRDR) of *rpoB* (Table 1); while rare, Mtb strains with RIF resistance-conferring mutations located outside the RRDR have been observed clinically and would be missed by this test [14]. The Hain LPAs come in two forms: one for resistance to first-line drugs (INH and RIF) and another for fluoroquinolones and the second-line injectable drugs [15]. A new version of Xpert MTB/RIF that detects common resistance-conferring mutations to INH, fluoroquinolones and aminoglycosides has also been developed [16]. While the LPAs can identify specific mutations, Xpert MTB/RIF infers the presence of mutations through the absence of the ‘wildtype’ and can give a resistance result in the presence of silent mutations [17]. These inconsistencies are also evident in genotypic tests to other first-line drugs and the major second-line drugs [18].

Box 2 **Phenotypic drug susceptibility testing.**

In culture-based DST, resistance is defined as the ability >5% of the Mtb population to grow at or above a pre-defined critical concentration (CC) of drug [9]. CC values for individual drugs are recommended by the WHO/Clinical and Laboratory Standards Institute (CLSI) and were originally defined by the WHO in 1969 as drug concentrations that were higher than those in which wildtype strains (`strains of the human type that have never come into contact with the drug’) could grow [8,9]. CC values are thus related to the distribution of MICs for clinical Mtb strains, and the highest MIC for strains that have no detectable resistance (genotypic or phenotypic, *i.e.,* wildtype) is defined as the epidemiological cut off (ECOFF) [9,24^••^,^26^]. The ECOFF is the lowest possible CC but, as discussed below, CCs are sometimes much higher than observed ECOFFs, which can lead to breakpoint artefacts [9,24^••^,^26^].

This complex and outdated definition of phenotypic susceptibility/resistance for TB is fraught with problems. For most drugs, only a single CC value is used for DST. This results in the binary classification of an Mtb sample as either resistant or susceptible, and precludes determination of the level of resistance associated with a particular sample [8]. Consequently, patients infected with Mtb strains that have low levels of resistance who may benefit from higher dosage of a drug would not be detected [8]. Moreover, breakpoint artefacts occur when the CC is higher than the ECOFF [9,24^••^]. This results in strains with MIC values that are higher than the ECOFF but lower than the CC being classified as susceptible, leading to the inclusion of a likely ineffective drug in a treatment regimen [9,24^••^].

Alt-text: Box 2

Given the complex genetics of TB drug resistance, it is unlikely that a single molecular diagnostic will be able to cover the full spectrum of mutations associated with the large number of drugs/drug classes that are used to treat DR-TB [19^•^] (Table 1). However, the logical extension of genotypic DST, enabled by technological advances and plummeting costs, and informed by whole-genome sequencing (WGS) of Mtb strain collections [20, 21, 22, 23], is to use WGS for routine diagnosis, drug resistance detection and strain typing, as implemented recently by Pubic Health England [24^••^,25^••^]. However, questions on whether and to what extent a genetic variant confers resistance, and what the clinical relevance might be, remain open for many new and existing TB drugs and will need to be addressed in order to realise the potential of this approach [19^•^,^26^]. The rapidly expanding databases that link genetic polymorphisms in Mtb associated with TB drug resistance with clinical metadata will be instrumental in this regard (Box 3).

Box 3 **Databases and online resources for tb drug resistance.**

A number of databases that catalogue known drug resistance-conferring mutations in Mtb have been developed. In addition, increased use of WGS to analyse large panels of drug-susceptible and drug-resistant strains has led to the development of several tools that can identify resistance mutations in raw sequencing reads. While some tools report sensitivity and specificity in terms of detecting drug resistance, none has yet been endorsed by the WHO for clinical use. Here, we briefly describe each resource and provide a URL, where available.

**Databases and tools available online**

**TBDreamDB** [67] (https://tbdreamdb.ki.se/Info/Default.aspx) was developed via a systematic review of literature describing drug resistance-conferring mutations in Mtb. Information about whether a particular mutation is observed more often in a DR rather than drug-susceptible strain is included, along with information describing which mutations are more commonly observed in association with resistance to particular drugs.

The **ReSeqTB** platform [26] (https://platform.reseqtb.org/) was specifically established to facilitate on-going development of a WHO-endorsed diagnostic assay for Mtb. The database sources WGS data, collects associated clinical and phenotypic metadata, and analyses all data according to a pre-defined pipeline. This database is actively curated as new information on TB drug resistance mutations becomes available. However, access to the database requires permission from the developers.

**PolyTB** [68] (http://pathogenseq.lshtm.ac.uk/polytblive/browser.php) was developed by Coll and colleagues, after processing raw WGS data for 1627 Mtb strains from publicly available datasets. The tool allows for manual searching of SNPs in any Mtb gene, as well as searching for SNPs in genes of interest such as those associated with drug resistance. This allows the user to gain a sense of how many strains within the collection contain a particular resistance mutation, and provides some information about the strain lineage and geographical area from which the strain was isolated.

**TB Profiler** [69^•^] (http://tbdr.lshtm.ac.uk/), another separate tool developed Coll and colleagues, allows input of any raw WGS data in fastq format and provides information about resistance to common TB drugs as well as the Mtb strain lineage.

The **PhyResSE tool** [70] (http://phyresse.org/) is another online tool that allows input of fastq files from Illumina-generated WGS data and provides information about drug resistance patterns and strain lineage.

**Downloadable tools**

**Mykrobe Predictor TB** [71] supports the input of raw WGS data generated on an Illumina platform to report mutations associated with drug resistance in Mtb. The tool can reportedly detect low frequency populations, which is one of the features that differentiates it from TBProfiler.

**KvarQ** [72] is another user-friendly tool that can rapidly detect mutations associated with drug resistance within raw WGS data. Information on strain spoligotype and lineage is also provided, and modules can be modified to detect user-specified mutations.

Alt-text: Box 3

## Heteroresistance — another complicating factor

**Figure 1:**
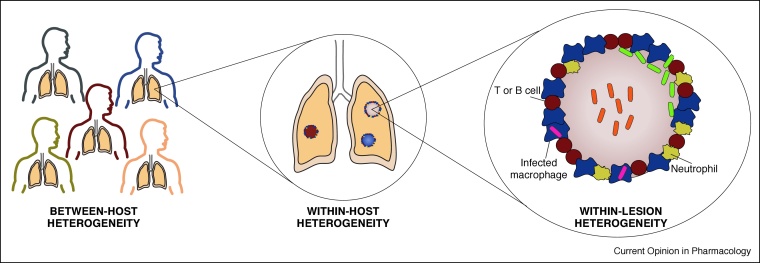
Heterogeneity in TB disease impacts the response to treatment. Heterogeneity is evident at multiple levels in TB disease [29^••^,^48^,^49^]. Differences in host genetics, immune status, co-infections and socioeconomic factors can impact susceptibility to TB infection and progression of disease. Once an individual becomes infected with Mtb and develops TB disease, the immune response and the response to TB chemotherapy can vary between TB lesions leading to differences in the kinetics of resolution between lesions, depicted here by different coloured lesions in the lung, and may result in the development of drug resistance in subpopulations of Mtb within distinct lesions [30].Within granulomas, spatial heterogeneity can result in drug gradients and metabolic changes in Mtb populations which differentially affect drug efficacy [50] and may result in phenotypic heterogeneity among populations of bacilli within a granuloma.

Further complicating the diagnosis and management of DR-TB is the phenomenon of heteroresistance, which refers to the co-existence of susceptible and resistant Mtb variants, or of multiple resistant strains with discrete resistance-conferring mutations, within a single specimen. Heteroresistance, which can arise as a result of infection with different strains of Mtb or through mutation within a clonal Mtb population, is found in 5.38% DR-TB cases, depending on the setting, the specific drug/s, and the method used to detect resistance [27^•^]. Next-generation WGS has revealed significant levels of micro-heterogeneity at drug resistance loci within an individual patient [20,21,28]. Minority variants (<1–5% of the population) have been shown to change in frequency throughout the course of infection suggesting that Mtb samples mutational space until the fixation of a particular mutation eventually occurs [2^••^,^20^]. Further insight has come from recent studies highlighting the within-host heterogeneity of TB disease at a lesional level [29^••^] and the implications thereof for the evolution of heteroresistance (Figure 1). By combining serial computed tomography scanning with WGS of sputum samples, Lui *et al.* found that anatomically discrete lesions in a MDR-TB patient showed heterogeneous responses to treatment which could potentially be explained by the presence of heterogeneous populations of Mtb showing different patterns of mutations at drug resistance loci [30]. Of the various factors that might affect the dynamics of within-host microevolution of Mtb, differential lesion penetration by drugs [31^••^] is likely to be a particularly important driver of sub-population-specific drug resistance.

At a practical level, culture of Mtb isolates before DST can mask heteroresistance within samples; this has important implications for phenotypic testing for drug resistance [32] (Box 2), and hence, adverse consequences for patient management, particularly if underlying drug resistance is not detected by conventional DST [27^•^]. Thus, the ability to detect minority variants before drug resistance becomes detectable by conventional DST, could avoid inappropriate first-line treatment and improve treatment outcome for DR-TB patients [27^•^].

## Public health consequences of simplified diagnosis and treatment for a complex disease

TB is a complex disease with the largest disease burden located in low resource settings with weak health care systems and consequently more limited diagnostic and treatment capacity. For this reason, public health approaches to TB have incorporated simplified and standardised diagnostic and treatment algorithms aimed at care delivery at the non-specialist levels of health care systems [33]. While these approaches have undoubtedly contributed to expanded access to care and saved many lives, resistance has emerged to all anti-TB drugs in widespread use [34]. Given the complexity of TB disease, standardised treatment regimens, with standardised dosing, delivered regardless of disease location and severity, lung pathology and comorbidities such as HIV infection, have likely contributed to resistance emergence [35,36].

Treatment of DR-TB is even more complex and yet a similar approach of using standardised regimens based on resistance testing to a few key drugs is a key mechanism for expanding access to diagnosis and treatment for DR-TB [37^••^,^38^]. Currently, the majority of patients treated for RR-TB are given second-line regimens based on a single genotypic RIF-resistance result rather than a full resistance profile for all first-line and second-line TB drugs [1]. This single genotypic result assumes that a range of mutations in the RRDR of *rpoB* all confer the same degree of resistance to RIF (Box 2), and overlooks the complexities in interpreting DST results described above.

These complexities in DST are not evident to most clinicians receiving a dichotomous resistant/susceptible result from the laboratory. Clinicians use these dichotomous results (often from a single specimen) to either prescribe a standardised second-line regimen or, less often in high-burden settings, design regimens based on a classification of drugs recommended by the WHO [38]. On the basis of the complexities of resistance testing, it may seem desirable to provide considerably more detail in laboratory reports of resistance testing; for example, reporting the presence of mutations that may only have a moderate impact on the drug’s MIC. Recent moves towards using WGS to provide full resistance profiles to all available drugs, in high-resource, low-burden settings, go some way towards this end. This notion aligns with the individualised approach to the management of XDR-TB advocated by van Soolingen and colleagues [39]. However, for high-burden settings with limited resources, such an approach risks placing the diagnosis and treatment of DR-TB in the realm of specialised medicine, and hindering much needed expanded access to diagnosis and treatment [37^••^].

So, given the complexities, should we be aiming to develop more sophisticated resistance testing approaches that take into account both genotypic and phenotypic resistance data [9], in addition to other factors such as bacillary burden? Such an approach would include determining the true MIC for drugs tested against the Mtb strain/s isolated from the patient, and thus guide not only the inclusion of particular drugs in a regimen, but also appropriate dosing. To date, available evidence suggests that low-level drug resistance associated with particular mutations can be overcome for drugs such as INH [40] and emerging data suggest that RIF dosages can also be increased [41]. Ideally, more detailed resistance testing would also detect heterogeneity directly from biological specimens, and therefore any underlying drug resistance that could emerge during treatment. However, to be feasible in many settings, new diagnostic approaches such as this would need to be automated and able to be conducted in, at least, decentralised laboratories. Advances in developing cheaper and higher throughput methods for MIC determination [42], for example, hold promise in this regard.

## How do we minimise future resistance emergence?

While poor adherence by patients has often been cited as the cause of TB drug resistance, evidence now suggests that factors such as individual pharmacokinetics [43], variable penetration of drugs into tuberculous lesions [31^••^] and use of standardised regimens in the presence of undiagnosed drug resistance may be primary drivers [44,45]. Greater degrees of treatment individualisation based on microbiological characteristics of the infecting bacteria (pre-existing resistance, heterogeneity, and strain type) and clinical characteristics of the patient might be expected to minimise the risk of further resistance emergence, particularly to new TB drugs and those in clinical development ([46]; https://www.newtbdrugs.org/). However, treatment individualisation requires an understanding of synergies, antagonism and cross-resistance for a wide range of possible combination regimens. A method developed recently for measuring higher-order drug interactions in Mtb *in vitro*, efficiently and at scale [47], may go some way towards addressing how TB drugs could be combined to produce shortened regiments that achieve durable cure and prevent the emergence of resistance. Ultimately, minimising further resistance while ensuring universal access to high quality care will require that innovative approaches that take the complexity of TB disease and drug resistance into account are developed and trialled in the settings in which they will be implemented.

## Conflict of interest statement

As member of an advisory group, V.M. has received an honorarium from the Bill and Melinda Gates Foundation. A.K. and H.C. have no conflicts of interest.

## References and recommended reading

Papers of particular interest, published within the period of review, have been highlighted as

• of special interest
•• of outstanding interest
